# Molecular mechanism of aberrant decidualization in adenomyosis leading to reduced endometrial receptivity

**DOI:** 10.3389/fendo.2024.1435177

**Published:** 2025-01-16

**Authors:** Yuanquan Dai, Zheng Yuan, Weisen Fan, Zhiheng Lin

**Affiliations:** ^1^ Department of Gynecology, Affiliated Hospital of Shandong University of Traditional Chinese Medicine, Jinan, Shandong, China; ^2^ Department of Gynecology, Guang anmen Hospital, China Academy of Chinese Medical Sciences, Beijing, China; ^3^ Department of Gynecology, Longhua Hospital, Shanghai University of Traditional Chinese Medicine, Shanghai, China

**Keywords:** adenomyosis, decidualization, stromal cells, endometrial receptivity, molecular mechanism

## Abstract

Patients with adenomyosis not only experience a decrease in quality of life as a result of dysmenorrhea and severe monthly flow but they are also rendered infertile. Pregnancy rates are still low among women with adenomyosis, even with assisted reproduction. According to the current study, endometrial receptivity is primarily responsible for the lower conception rate among patients with adenomyosis. Decidualization of endometrial stromal cells is the fundamental requirement for endometrial receptivity and the maintenance of a normal pregnancy, even though endometrial receptivity is made up of a variety of cells, including immune cells, endometrial epithelial cells, and endometrial stromal cells. Our overview reveals that endometriosis deficiencies are present in patients with adenomyosis. These flaws may be linked to aberrant pathways in endometrial stromal cells, such as PI3K/Akt, JAK2/STAT3, and hedgehog. Correcting the abnormal expression of molecules in endometrial stromal cells in the endometrium of patients with adenomyosis may become the focus of research to improve endometrial receptivity and increase the pregnancy rate.

## 1 Introduction

Adenomyosis of the uterus is more common in women between the ages of 30 and 50 who are fertile. It is frequently accompanied by infertility, increased menstrual flow, more prolonged menstruation, and progressive dysmenorrhea. Patients may experience hemorrhagic shock in cases where the illness is more severe (1). Patients with Adenomyosis have long-term menstrual blood loss and dysmenorrhea symptoms, which impair their quality of life and harm their physical and emotional health. Statistics show that Adenomyosis can have a prevalence of up to 70% and an incidence of 7% to 23% (2). The incidence of Adenomyosis was found to be 20.0% in infertile patients under 40 years old and 29.7% in those over 40 years old in a cross-sectional study of infertile individuals evaluated by transvaginal 3D ultrasonography (3). Of the women who need assisted reproductive technologies, between 30% and 40% have adenomyosis (4). Adenomyosis also exhibits the phenomena of delayed diagnosis in its early stages. It has the traits of a chronic illness, meaning that not only will the symptoms worsen over time, but it will also be challenging to treat (5). Consequently, Adenomyosis seriously threatens national population security, public health, and social advancement.

Patients with adenomyosis are currently treated with both surgical and drug-conservative methods. However, people with adenomyosis who are infertile frequently require the assistance of assisted reproductive technology (6). Even though individuals with adenomyosis can donate eggs and acquire embryos for transfer, their low endometrial reactivity frequently results in a lower rate of embryo implantation and a higher risk of premature termination (7). Due to adenomyosis, some individuals may experience many obstetric difficulties, including preterm birth, even after a good pregnancy (8). The complicated process underlying the lower embryo implantation rate in adenomyosis patients is mainly associated with abnormal endometrial decidualization, structural and functional alterations in the endometrial-myometrial junction, and abnormal endometrial microenvironment (9). Normative embryo development and decidualization of the endometrium are the two most essential phases in the human reproductive process. Any issues with these processes could result in an abnormal pregnancy or perhaps the pregnancy failing outright (10). Consequently, to improve spontaneous conception and assisted reproductive treatment of adenomyosis-related infertility, it is critical to elucidate the mechanism of decreased endometrial receptivity induced by aberrant decidualization of endometrium in adenomyosis.

## 2 Related cells involved in the formation of endometrial receptivity

During the secretory phase, the endometrium can undergo many morphological and functional changes and receive and accept embryos. This process is known as endometrial receptivity. Usually occurring 7-10 days following ovulation, the window of implantation(WOI) is the best time for the endometrium to receive embryos during the secretory phase. Under the influence of hormones, the endometrial stromal cells, immune cells, and endometrial epithelial cells contribute to establishing receptivity.

### 2.1 Endometrial epithelial cells

The endometrial epithelial cells are the first stage of embryo implantation into the uterus. Endometrial epithelial cells may use tumor necrosis factor-alpha converting enzyme/A Disintegrin And Metalloprotease-like during the secretory phase of the endometrium to decrease the expression of mucin 1 (11). Mucin 1 can shield the endometrium from bacterial and protease damage. Moreover, it can stop cell adhesion factors from being exposed, which is counterproductive to cell interaction (12). Usually, mucin 1 is downregulated in the embryo implantation area, especially in the exocytosis structure of endometrial epithelial cells during the secretory phase, where there is almost no attachment of mucin 1 (13). At the same time, the adhesion factor L-selectin and its ligand are expressed on the surface of embryonic trophoblast cells and endometrial epithelial cells during the WOI, respectively (14). L-selectin is a component of the embryonic endometrial junction and could represent the initial stage of the mother-fetal junction (15). Furthermore, endometrial epithelial cells feature integrins on their surface that function as connectors with the extracellular matrix(ECM). When the endometrium is in the middle stage of secretion, integrin can form a composite structure of “integrin ECM integrin” or “ECM integrin” with the extracellular matrix, which helps with the connection between mother and fetus (16). During the WOI, endometrial epithelial cells proliferate slowly and depolarize under the action of progesterone, which also provides conditions for further embryo implantation into the uterus. Progesterone may inhibit endometrial epithelial cell proliferation by a mechanism that involves upregulating the expression of Kruppel-like factor 15 and Bone morphogenetic protein receptor type-1A (17, 18). The depolarization of endometrial epithelial cells is mainly manifested by a decrease in intercellular connections and a loosening of intercellular spaces. Endometrial epithelial cells’ depolarization is mainly determined by Homeobox protein MSX(Msx), which inhibits Protein Wnt-5a to reduce the expression of these cells’ E-cadherin/catenin complex (19). An essential element of the link between endometrial epithelial cells is the E-cadherin/catenin complex. Endometrial epithelial cells will continue to express E-cadherin as Msx expression declines, which is detrimental to embryo implantation (20).

### 2.2 Endometrial stromal cells

Effective decidualization of the endometrium indicates good responsiveness. While several types of cells are involved in decidualization, endometrial stromal cells are the predominant kind. Endometrial stromal cells migrate outward from the implantation site like endometrial epithelial cells, creating an embryonic penetration pathway (21). Endometrial stromal cells undergo a decidualization reaction after 5-6 days of fertilization. Endometrial stromal cells transform from spindle-shaped to circular, characterized by increased cell volume, cytoplasmic expansion, and accumulation of glycogen and lipid droplets (22). The dense outer layer of the decidua contains decidualized endometrial stromal cells that can secrete prolactin(PRL) and insulin-like growth factor binding protein 1(IGFBP-1), which can stimulate trophoblast cell infiltration and proliferation, control the survival of natural killer cells in the endometrium, and encourage angiogenesis (23). Of course, the invasion of embryos is not unlimited. Decidualized endometrial stromal cells also prevent trophoblasts from invading to regulate the extent of embryo implantation. To prevent extracellular trophoblast invasion, the stromal cells in the decidua’s dense layer create extracellular matrix proteins such as fibronectin, laminin, and heparin sulfate proteoglycans (24).

Progesterone and 3’,5’-Cyclic AMP(cAMP) at high levels are key beginning signals that mediate the decidual response (25), while Peptidyl-prolyl cis-trans isomerase FKBP4 and Steroid receptor coactivator 2 are crucial cofactors that initiate signal transduction (26, 27). Usually, cAMP is engaged in the process of stopping a cell’s reaction to long-lasting outside stimuli. To maintain a decidualized phenotype, human endometrial stromal cells require continuous stimulation of the cAMP pathway (28). CAMP activation requires progesterone to bind to progesterone receptors, which bind to downstream Protein kinase A(PKA) and activate and release catalytic subunits on PKA, which phosphorylate cyclic AMP-responsive element-binding protein(CREB) and cAMP response element modulator(CREM) (29). Activated CREB and CREM can recruit CREB-binding protein, which synergistically regulates the expression of progesterone receptors with Steroid receptor coactivator 1, thereby increasing the sensitivity of endometrial stromal cells to progesterone (30). CAMP/PKA regulates Pleiotrophin(PTN) expression, and PTN expression can encourage Prolactin family 8, subfamily a, member 2(PR18A2) and Prolactin family 3, subfamily c, member 1(PR13C1) expression. Endometrial stromal cells are regulated by PR18A2 and PR13C1, which are crucial for their proliferation. CAMP/PKA regulates PTN expression, and PTN expression can encourage PR18A2 and PR13C1 expression. Endometrial stromal cells are regulated by PR18A2 and PR13C1, which are crucial for their proliferation (31).

Bone morphogenetic protein 2(BMP2) is a crucial regulatory element that encourages uterine stromal cells to decidualize. Although the precise process is still unknown, cAMP/PKA may also be used by BMP2 to promote decidualization (32). Progesterone can cause Indian hedgehog protein(IHH), which controls COUP transcription factor 2 expression and, in turn, BMP2 (33). BMP2 can directly regulate FK506-binding proteins to promote the decidualization of stromal cells and promote the activity of progesterone receptors (34). By triggering Protein Wnt-4(Wnt4), BMP2 can also control the expression of Forkhead box protein O1(FOXO1). At the start of decidualization, substantial amounts of FOXO1 reach the nucleus. Before binding to gene enhancers, FOXO1 can interact with Homeobox protein Hox-A10(HOXA10) and CCAAT/enhancer binding protein and promote the production of the markers PRL and IGFBP1, which speeds up the decidualization process (29, 35). BMP2 inhibition is caused by Krueppel-like factor 9 upstream in the absence of decidualization. Following decidualization, the inhibition is released, and the effects of BMP2 start (36). Amine oxidase [flavin-containing] A(MAOA) can also promote FOXO1. When MAOA expression decreases, FOXO1 expression decreases, abnormal proliferation of endometrial stromal cells occurs, and endometrial receptivity decreases (37). Of course, numerous studies have also demonstrated that controlling progesterone receptor expression and activity can control the decidualization process. For example, phosphoinositide-3-kinase regulatory subunit alpha(PIK3R1) is a cofactor of progesterone receptor transcription. Knocking down PIK3R1 reduces the expression levels of FOXO1 and Wnt4, affecting endometrial cell proliferation and differentiation (38). Polycomb complex protein BMI-1 and progesterone receptors can interact, regulate progesterone receptor ubiquitination, and maintain normal progesterone receptor-hormone responses (39). Lack of Endothelial transcription factor GATA-2(GATA2) can lead to decreased expression of progesterone receptors and weakened progesterone signal transduction. GATA2 not only participates in the expression of progesterone receptors but also co-regulates downstream progesterone response genes with progesterone receptors (40).

**Figure 1:**
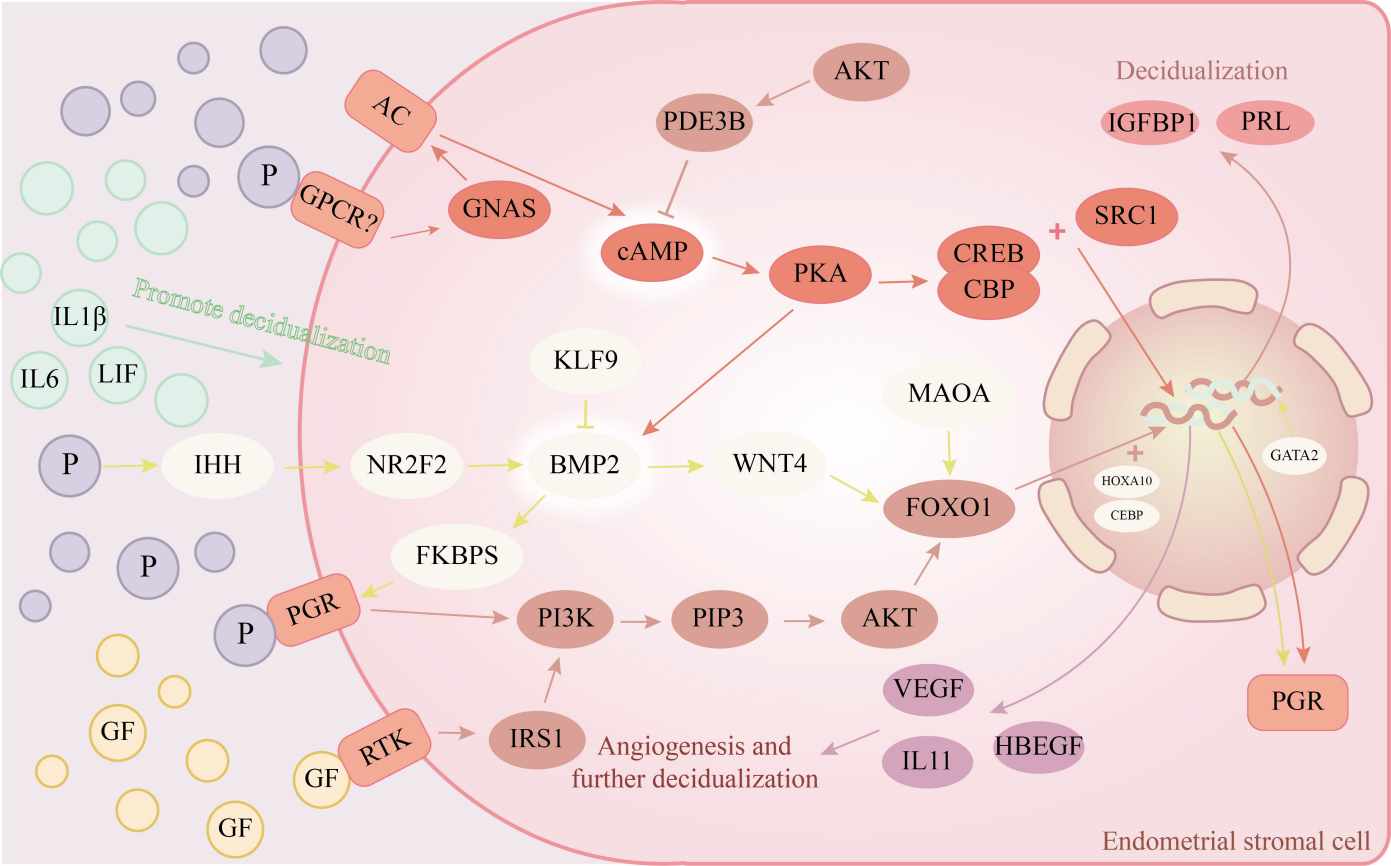
Endometrial stromal cells’ decidualization molecular pathway diagram. →represents promotion, ┨represents inhibition. The rounded rectangle in the illustration represents the receptors on the cell membrane; Circles show hormones or extracellular substances, while ellipses represent different cell targets.

There is also some infectiousness to the decidualization of stromal cells. Endometrial stromal cells can increase the decidua response by producing autocrine or paracrine cytokines, such as Proheparin-binding EGF-like growth factor(HB-EGF), activin, and Interleukin-11(IL-11) after the decidua is finished (41–43). IL-11 receptor-deficient mice cannot undergo stromal decidualization, and while embryos can be implanted, early pregnancy loss happens (44). Blocking HB-EGF signaling can cause stromal cell death, increase the pro-inflammatory pathway inside endometrial stromal cells, and prevent endometrial stromal cells from decidualizing (45). In addition to the regulatory effects of stromal cells, epithelial cells and embryonic trophoblast cells can also secrete Leukemia inhibitory factor(LIF) and Interleukin-1β、 Extracellular factors such as Interleukin-6(IL-6) further induce the decidualization of stromal cells (46, 47). Endometrial stromal cells also play a role in vascular remodeling during decidualization. During decidualization, endometrial stromal cells also take part in vascular remodeling. Progesterone and cAMP combined *in vitro* can also cause Vascular endothelial growth factor expression during decidualization (48). Extracellular vesicles produced by endometrial stromal cells can promote endothelial cell proliferation and angiogenesis (49). The molecular mechanism of endometrial stromal cell decidualization can be seen in **Figure 1**.

### 2.3 Immune cells

Immune cells can facilitate embryo implantation and development by collaborating with endometrial stromal and epithelial cells. The immune cells primarily engaged in endometrial receptivity are T cells, dendritic cells, natural killer cells, and macrophages. Among them, natural killer cells account for 70% of uterine immune cells. Peripheral recruitment and precursor differentiation may be the source of uterine natural killer cells (50, 51). In terms of function, natural killer cells in the uterus differ from peripheral natural killer cells that dissolve tumor cells. Uterine natural killer cells contain lysozyme and granulosa but have no cytotoxicity (10, 52). One of the critical factors in fetal development and the formation of the placenta is believed to be the interaction between Killer-cell Immunoglobulin-like Receptor(KIR) on natural killer cells and HLA class I histocompatibility antigen, C alpha chain(HLA-C) on trophoblast cells. Certain combinations of KIR and HLA-C can lead to unfavorable pregnancy outcomes, like fetal growth limitations and recurrent miscarriages, especially in mothers expressing exclusively inhibitory KIR and babies with high expression of HLA-C2 (53, 54). The absence of natural killer cells in the placenta of rats results in a hypoxic environment, and the trophoblast cells outside the villi show greater invasiveness. These findings suggest that natural killer cells can control vascular remodeling, affecting the trophoblast cells’ capacity to invade the endometrium (55). In the endometrium of an early pregnancy with decidualization, there are a lot of uterine natural killer cells. They can cause the extracellular matrix to break down and vascular smooth muscle cells to be destroyed, suggesting that natural killer(NK) cells may be engaged in the early spiral artery remodeling process (56).

Approximately 15% to 20% of white blood cells in the uterine decidua are macrophages. Uterine macrophages primarily migrate toward the M1 type during non-pregnancy. Following placental development during pregnancy, macrophages will change to the M2 phenotype and stay there until delivery (57). Decidual macrophages, like natural killer cells, are found in and around trophoblast cells and spiral arteries. They are involved in trophoblast cell infiltration, spiral artery remodeling, and embryo implantation (58). Decidual macrophages continuously eliminate apoptotic cells throughout pregnancy to stop apoptotic bodies from releasing and triggering a decidual inflammatory response. This procedure will strengthen the immunosuppressive milieu during pregnancy by encouraging macrophages to secrete more immunosuppressive substances (59, 60). Antigen-presenting solid cells that activate effector T cells to induce cellular immunological responses can be produced by dendritic cells. Immature dendritic cells can improve immunological tolerance by encouraging the production of Treg cells that have undergone differentiation (61). Oddly, throughout pregnancy, dendritic cells steadily decline (62). Moreover, Bartmann et al. discovered that the majority of the dendritic cells in the decidua are immature dendritic cells (63). This might be the decidua’s immune system being suppressed during embryo implantation. Treg cells are essential in mediating maternal tolerance to allogeneic fetuses during implantation and early pregnancy (64). Clinical studies have demonstrated that the presence of Treg cells can lessen the likelihood of unfavorable pregnancy outcomes (65). T helper 2 cell(Th2) predominantly secretes transforming growth factor-β, Interleukin-4, Interleukin-5, and Interleukin-10. Await cytokines facilitate humoral immunity, reduce the activation of the immune system, and encourage the development of allogeneic immunological tolerance. The primary function of T helper 1 cell(Th1) is the production of extracellular cytokines, including IL-2, TNF-α, and IFN-γ, which are detrimental to embryo implantation because they engage in immune surveillance, prevent trophoblast invasion, and activate NK cells (66). The Th1/Th2 balance at the mother-fetal interface is critical in sustaining pregnancy under normal conditions. Treg cells maintain the interplay between Th1 and Th2 cells. In normal pregnancy, this balance will favor the Th2 type (67).

## 3 Patients with adenomyosis have decreased endometrial receptivity

In addition to the endometrium’s decreased receptivity, altered uterine cavity anatomical morphology may contribute to the lower embryo transfer success rate in adenomyosis patients. Nonetheless, the lower endometrial receptivity in adenomyosis patients is a fundamental obstacle to embryo implantation. Concerning embryo implantation, the aberrant development of embryos in the uterine cavity during the window period, the aberrant contact between endometrial epithelial cells and embryos, and the abnormal endometrial decidualization are the main manifestations of the decrease in endometrial receptivity in adenomyosis. A specific concentration of free radicals in the uterine cavity environment is necessary for embryo implantation, and high or low levels are not conducive to embryo development and implantation (68). The menstrual cycle’s changes will affect the free radical-regulating enzymes Xanthine oxidase (XO), Superoxide dismutase (SOD), Glutathion peroxidase, and Nitric oxide synthase (NOS). The quantities of NOS, XO, SOD, and catalase in the endometrial epithelium, however, are overexpressed in patients with adenomyosis and do not change over the menstrual cycle (69–72). However, the damaging mechanism of endometrial oxidase and antioxidant enzyme expression disorders in embryos in adenomyosis has not yet been studied. Integrin β3 is an essential protein for the attachment of embryos to endometrial epithelial cells and a marker molecule that can indicate the endometrium’s receptivity. A tiny integrin ligand called osteopontin can attach to integrins β 3. It can also control how trophoblast and endometrial epithelial cells interact during implantation. However, integrin β3 and osteopontin expression in endometrium decreased during the WOI of adenomyosis (73). L-selectin might be the initial protein implicated in the endometrial-embryonic junction. Similarly, throughout the window period of adenomyosis, its expression declines in the endometrium (74). Reduced interaction between endometrial epithelial cells and embryos is shown by the decreased expression of protein molecules representing the contact between endometrial epithelial cells and embryos in patients with adenomyosis.

Adenomyosis patients’ decidualization differs significantly from that of healthy women, and aberrant decidualization can result in unsuccessful embryo implantation (75). Endometrial stromal cells from patients with adenomyosis have been found to display decidualization abnormalities *in vitro* (76). The hormone-dependent junction zone(JZ) between the basal layer and inner layer of the endometrium can regulate the peristalsis of the uterine and endometrial layers. The amplitude and frequency of peristaltic waves gradually increase with the release of luteinizing hormone (77). JZ’s thickness and contraction can impact the transport and implantation of developing embryos. It is linked to the development of the decidua and endometrium and the remodeling of spiral arteries (78). An indication of endometrial receptivity of adenomyosis is the thickness of JZ. The success rate of embryo suppression is 45% when the thickness of JZ is less than 10 mm, while the pregnancy rate is only 5% when the thickness of JZ is more significant than 12 mm (79). Regular expression of estrogen and progesterone receptors in the endometrium is necessary for the endometrial in women of reproductive age to remain receptive. However, progesterone receptor expression declines in the endometrium of individuals with adenomyosis (80). Endometrial decidualization requires the binding of progesterone receptors to progesterone, particularly during pregnancy when endometrial stromal cells express significant levels of progesterone receptors (81). Consequently, because progesterone receptors are absent in people with adenomyosis, the endometrium may abnormally decidualize, decreasing endometrial receptivity and making it difficult for embryos to pierce deeply into the endometrium.

## 4 Molecular mechanism of abnormal decidualization of stromal cells in adenomyosis of the uterus

**Figure 2:**
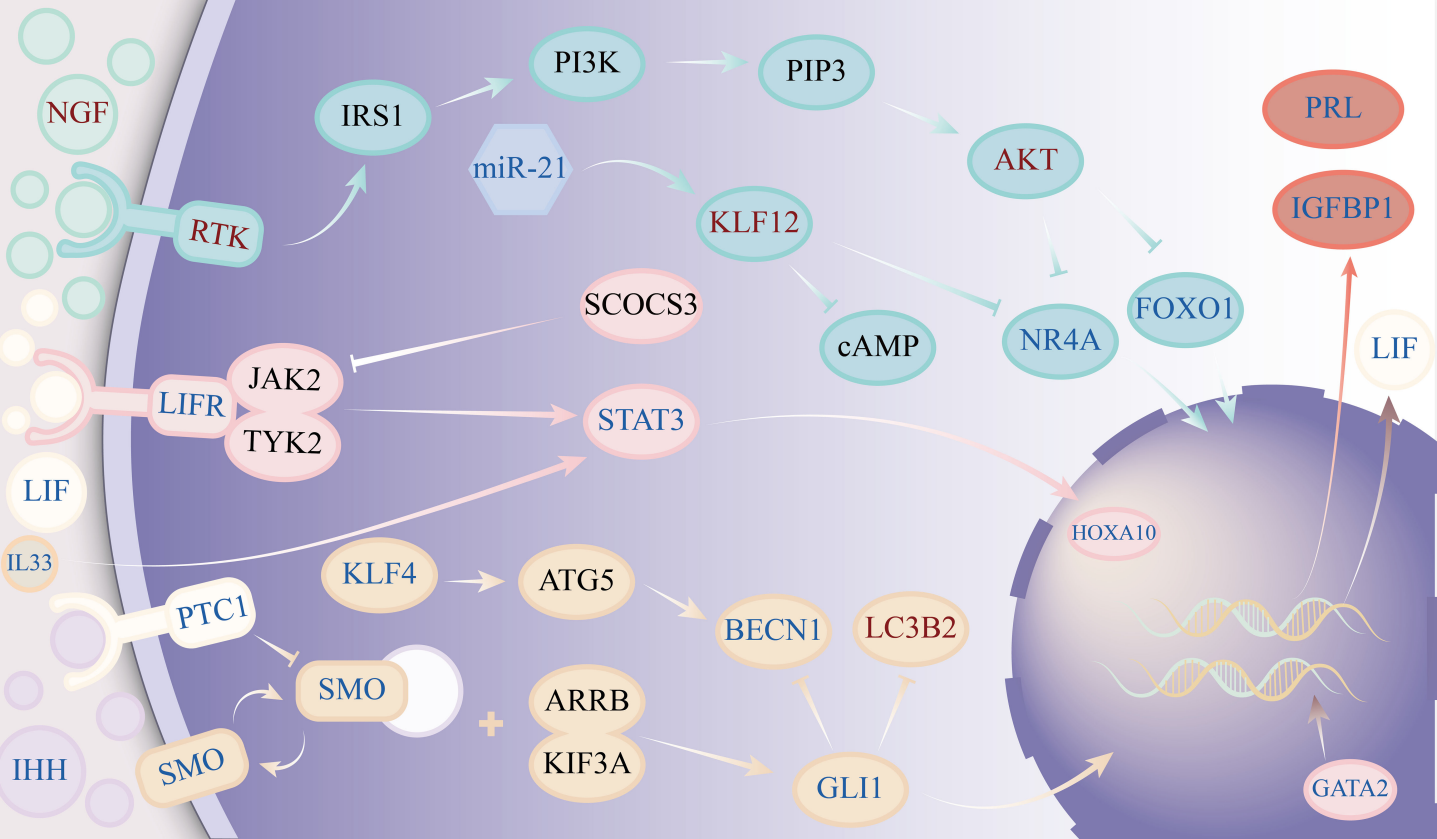
Diagram showing the molecular mechanisms underlying the aberrant decidualization of endometrial stromal cells during adenomyosis. The figure’s dark text signifies that further confirmation is needed to confirm the target’s anomaly. Red symbols indicate an increase in expression, whereas blue characters indicate a decrease in expression.

According to clinical and basic research findings, adenomyosis patients have inadequate decidualization of endometrial stromal cells. While the exact cause of abnormal decidualization of endometrial stromal cells in adenomyosis patients remains unknown, our investigation of the molecular mechanism of abnormal decidualization of endometrial stromal cells in adenomyosis primarily reveals abnormalities in pathways such as phosphatidylinositol 3-kinase/protein kinase B(PI3K/Akt), Janus kinase 2/signal transducer and activator of transcription 3(JAK2/STAT3), and hedgehog. The detailed molecular mechanism of abnormal decidualization of endometrial stromal cells in adenomyosis can be seen in **Figure 2**.

### 4.1 PI3K/AKT pathway

Numerous cytokines can activate the PI3K/Akt signaling pathway, which controls transcription, translation, and cell survival. Following PI3K activation by different cytokines, PI3K catalyzes the synthesis of 3,4,5-triphosphate phosphatidylinositol, which therefore causes Akt phosphorylation. The majority of the time, the phosphorylated Akt shows inhibitory regulation of several cellular processes (82). PI3K/AKT is activated in adenomyosis ectopic lesions, which facilitates the growth of ectopic endometrial cells (83). In patients with adenomyosis, phosphorylated AKT is also abundantly expressed in the eutopic endometrium. Nuclear receptor subfamily 4 group A member(NR4A) can upregulate the expression of FOXO1, while phosphorylation of AKT can result in a decrease in the levels of both NR4A and FOXO1. By upregulating the expression of PRL and IGFBP-1, NR4A can control the decidualization of endometrial stromal cells *in vitro* (75). As was previously established, FOXO1 plays a significant role in the decidualization of endometrial stromal cells. Irregularities in the AKT/NR4A/FOXO1 pathway may be the reason behind aberrant decidualization in endometrial stromal cells during adenomyosis. Yamamoto (84) et al. discovered that estrogen-stimulated stromal cells in adenomyosis lesions could create more Nerve growth factor(NGF) and that NGF could activate the PI3K/AKT pathway by stimulating Probable serine/threonine-protein kinase RTK1/Insulin receptor substrate 1 (85, 86). Increased expression of BDNF/NT-3 growth factors receptor(NTRK2) in endometrial tissue of patients with adenomyosis during secretion phase (87). Consequently, the excessive generation of NGF in the lesion and the binding of NTRK2 in stromal cells may be connected to the activation of PI3K/AKT in endometrial stromal cells during adenomyosis. Yan et al (88) discovered that Krueppel-like factor 12(KLF12) could prevent PI3K/AKT/NR4A-induced decidualization of endometrial stromal cells. According to this study, KLF12 expression was up in the endometrium of patients with adenomyosis, whereas the expression of miR-21 and NR4A was decreased in comparison to normal female endometrium. MiR-21 can suppress KLF12 expression, which in turn suppresses NR4A expression. Additionally, endometrial stromal cells’ cAMP level and decidualization can be inhibited by the high expression state of KLF12. This shows that by controlling KLF12 to suppress the PI3K/AKT/NR4A pathway and cAMP expression in stromal cells, miR-21 may prevent the onset of endometrial stromal decidualization.

### 4.2 JAK2/STAT3 pathway

The primary transduction mechanism of many cytokines and growth factors is the JAK2/STAT3 signaling pathway, primarily triggered by interleukins, tumor necrosis factor, and epidermal growth factor (89). When various cytokines bind to homologous receptors, JAK2 in the tyrosine kinase family is activated, causing STAT3 to be phosphorylated, forming dimers and interferon regulatory factors that enter the nucleus, thereby regulating the expression of target genes related to proliferation, apoptosis, and differentiation (90). Curiously, though, patients with adenomyosis display distinct JAK2/STAT3 pathway states in various tissues. In general, the formation of adenomyosis lesions involves the activation of JAK2/STAT3, and the development of adenomyosis lesions can be inhibited by blocking this route (91, 92). However, phosphorylated STAT3 activation can control the endometrium’s receptivity and decidualization, which is essential for successful embryo implantation and continued growth. The decidualization of human endometrial stromal cells shows intense p-STAT3 staining in the late stage of cell cycle secretion, and phosphorylated STAT3 is at a high level in stromal cells of naturally pregnant mice compared to those of nonpregnant mice (93, 94). The expression of PRL and IGFBP-1, indicators of the decidualization of endometrial stromal cells, was downregulated when the JAK2/STAT3 pathway was inhibited (95). Interestingly, the endometrium of patients with adenomyosis exhibits an inhibitory state of this pathway. This could be because the endometrial expresses fewer different cytokines and receptors. For example, Yen CF et al (96) found that the expression of LIF receptors in the eutopic endometrium of patients with adenomyosis decreased, and the activation of STAT3 and ERK signaling pathways was significantly reduced. The reduced expression of GATA2, which causes progesterone receptor expression in endometrial stromal cells of patients with adenomyosis, may be connected to the poor expression of LIF (97). The STAT3 pathway is strongly stimulated when endometrial stromal cells are cultured with LIF (96). Patients with adenomyosis have decreased expression of Interleukin-33(IL-33) in their endometrium. An increase in IL-33 expression can promote the phosphorylation of STAT3, increase the expression of HOXA10, and increase the embryo implantation rate (98). This points to a reduction in IL-33 in the endometrium, which might inhibit endometrial stromal cells’ ability to complete decidualization by silencing the JAK2/STAT3 pathway in these cells. Moreover, IL-6, IL-11, and Interleukin-27 might encourage the decidualization of endometrial stromal cells by activating the JAK2/STAT3 pathway (47, 99, 100). Overexpression of Suppressor of cytokine signaling 3 can prevent cytokine activation of the JAK2/STAT3 pathway by inhibiting STAT3 phosphorylation (95).

### 4.3 Hedgehog pathway

The hedgehog pathway, which involves primary ciliary transduction, regulates cell proliferation, tissue patterning, stem cell maintenance, and development in many tissues. Furthermore, progesterone signaling transmission is linked to this route (101, 102). Decidualization requires an increase in the quantity and length of primary cilia in uterine stromal cells, which occurs in the early stages of pregnancy. Progesterone has been shown to stimulate the cilia and the synthesis of IHH and Sonic hedgehog protein(SHH) in endometrial stromal cells in both *in vitro* and *in vivo* investigations. Through pathways reliant on IL-11 and primary cilia, SHH stimulates traditional Hedgehog signaling in stromal cells and encourages decidualization (103). The expression of the decidualization markers BMP2 and Wnt4 in the endometrium is downregulated when the expression of the IHH receptor PTCH1 declines (104). Zhou Y et al. discovered that in the endometrial tissue of individuals suffering from adenomyosis, there was a downregulation of the expression of pertinent targets in the Hedgehog pathway. Autophagy rises in endometrial stromal cells when the Hedgehog pathway is inhibited (105). Mei et al. discovered, however, that the decidualization of endometrial stromal cells rose in response to an increase in their autophagy function (106). Krueppel-like factor 4(KLF4), which has been shown to be downregulated in adenomyosis endometrial tissue, governs this process. It is the JAK2/STAT3 pathway that regulates KLF4 upstream (107). The downregulation of the JAK2/STAT3 pathway corresponded with the low expression of KLF4 in the endometrial tissue of adenomyosis. However, what confuses us is that the research results of Zhou Y et al. indicate that autophagy levels in the endometrium of patients with adenomyosis are enhanced (105). The research conclusions of the two are contradictory. Therefore, more research is required to determine if autophagy in the endometrial stromal cells of individuals with adenomyosis enhances the decidualization of endometrial stromal cells and the mechanism of its effect.

## 5 Conclusions

The last stage of female pregnancy is good endometrial receptivity, which is mostly associated with endometrial stromal cells, endometrial epithelial cells, and different immune cells. Building endometrial receptivity and sustaining a normal pregnancy require a fully functional endometrial decidualization response. Decidualization is a highly regulated physiological step that is intricate and mostly associated with major targets like cAMP, FOXO1, BMP2, and HOXA10. According to our analysis, endometrial decidualization problems in patients with adenomyosis are brought on by aberrant pathways in the endometrial stromal cells of adenomyosis, including PI3K/Akt, JAK2/STAT3, and hedgehog. The mechanism of aberrant endometrial decidualization in patients with adenomyosis resulting in long-term pregnancy diseases, as well as the function of different immune cells in the process of endometrial stromal cell decidualization, remain unclear and are not well documented in case studies. The molecular mechanism by which aberrant endometrial decidualization in adenomyosis causes a reduction in endometrial receptivity is clarified by this work, in summary. This can offer focused therapy regimens for the decline in the rate of embryo implantation brought on by aberrant decidualization. Additionally, it can establish a basis for investigating the mechanism of disorders associated with prolonged pregnancy that result from aberrant endometrial decidualization in adenomyosis.
